# Transcriptional frontloading contributes to cross‐tolerance between stressors

**DOI:** 10.1111/eva.13142

**Published:** 2020-10-22

**Authors:** Michael Collins, Melody S. Clark, John I. Spicer, Manuela Truebano

**Affiliations:** ^1^ Marine Biology and Ecology Research Centre, School of Biological and Marine Sciences University of Plymouth Plymouth UK; ^2^ British Antarctic Survey Natural Environment Research Council Cambridge UK

**Keywords:** cross‐tolerance, environmental change, frontloading, multistressor, plasticity

## Abstract

The adaptive value of phenotypic plasticity for performance under single stressors is well documented. However, plasticity may only truly be adaptive in the natural multifactorial environment if it confers resilience to stressors of a different nature, a phenomenon known as cross‐tolerance. An understanding of the mechanistic basis of cross‐tolerance is essential to aid prediction of species resilience to future environmental change. Here, we identified mechanisms underpinning cross‐tolerance between two stressors predicted to increasingly challenge aquatic ecosystems under climate change, chronic warming and hypoxia, in an ecologically‐important aquatic invertebrate. Warm acclimation improved hypoxic performance through an adaptive hypometabolic strategy and changes in the expression of hundreds of genes that are important in the response to hypoxia. These ‘frontloaded’ genes showed a reduced reaction to hypoxia in the warm acclimated compared to the cold acclimated group. Frontloaded genes included stress indicators, immune response and protein synthesis genes that are protective at the cellular level. We conclude that increased constitutive gene expression as a result of warm acclimation reduced the requirement for inducible stress responses to hypoxia. We propose that transcriptional frontloading contributes to cross‐tolerance between stressors and may promote fitness of organisms in environments increasingly challenged by multiple anthropogenic threats.

## 1 INTRODUCTION

Animals in the wild naturally encounter changes to multiple environmental drivers (Boyd et al., 2018). In highly dynamic environments, such as estuaries, simultaneous or sequential changes to environmental factors are common (Gunderson et al., 2016). In addition to natural environmental fluctuations, free‐living wild animals across ecosystems are being increasingly pressured by greater intensities of stressor combinations under climate change, driving reductions in performance and fitness (Deutsch et al., 2015; Gunderson et al., 2016) Therefore, plasticity displayed upon exposure to one environmental driver may only be adaptive if it does not come at the cost of impaired performance under another, a phenomenon referred to as “cross‐susceptibility” (Todgham & Stillman, 2013). Trade‐offs between competing physiological demands from multiple stressors constrain fitness (Kelly et al., 2016). Those species which experience “cross‐tolerance,” where plasticity under one stressor enhances performance under others (Todgham et al., 2005), are likely to be more resilient to the naturally variable abiotic conditions experienced in the wild (Todgham & Stillman, 2013). Cross‐tolerance can arise from shared mechanisms elicited by stressors (Todgham & Stillman, 2013). However, stressor combinations have often been applied acutely and simultaneously which excludes the possibility for preparative mechanisms to arise (Gunderson et al., 2016; Todgham et al., 2005). A greater understanding of the mechanistic molecular basis for interactions between stressors is essential in order to accurately predict the capacity of organisms to acclimatize to altered multifactorial environments (Todgham & Stillman, 2013).

“Transcriptional/constitutive frontloading” (Barshis et al., 2013) has been recently proposed as an important mechanism in promoting the resilience of species under varying abiotic conditions (Barshis et al., 2013). Frontloading involves long‐term changes to constitutive gene expression which prepares for frequently encountered environmental stress and reduces the requirement for inducible stress responses (Barshis et al., 2013; Palumbi et al., 2014). Investigations of transcriptional frontloading to date have been restricted to understanding its consequences for performance under single stressors (usually temperature) (Barshis et al., 2013; Dong et al., 2008). Transcriptional frontloading of protective groups of genes such as cellular defences or metabolic genes has been demonstrated between populations along environmental gradients with consequences for whole‐organism thermal tolerance (Barshis et al., 2013; Dong et al., 2008; Kenkel et al., 2013). Frontloading has not yet been investigated in a multistressor context but could have significant fitness implications for species inhabiting highly dynamic environments. Given that transcriptional frontloading can arise from acclimatory effects (Palumbi et al., 2014), there is potential for frontloading during acclimation to one stressor to alter performance under another.

This study aimed to investigate whether transcriptional frontloading generates cross‐tolerance between stressors. We focussed upon the combination of thermal acclimation and hypoxia, given they have interactive physiological effects through aerobic metabolism, creating the potential for interactions at the cellular level (McBryan et al., 2013). Broadly, thermal acclimation could be predicted to alter hypoxic performance if it induces mechanisms to either increase oxygen supply or reduce oxygen demand, i.e. hypometabolism (Anttila et al., 2015; McBryan et al., 2013). Hypometabolism is a key response to reduce energy expenditure and prolong survival under periods of adverse abiotic conditions in a range of taxa (Larade & Storey, 2002).

The estuarine amphipod *Echinogammarus marinus* was utilized as a model for invertebrate species inhabiting dynamic multifactorial environments. *E. marinus* is abundant in estuarine mudflats and intertidal zones of the north‐east Atlantic (Lincoln, 1979) where it is a key mesograzer of algal material, both predator and prey to several macroinvertebrate species, as well as being prey for fish (Alexander et al., 2012; Beermann et al., 2018; Dick et al., 2005). Given the pivotal role of gammarid amphipods in coastal food webs, they are established sentinel species in ecotoxicology (Cogne et al., 2019; Gismondi & Thomé, 2016) and recent studies have also begun to investigate their genomic responses to climate change (Axenov‐Gribanov et al., 2016; Collins et al., 2019). In this study, *E. marinus* was acclimated under normoxic conditions at 10°C (cold acclimated: control) or 20°C (warm acclimated) for 7 days. Following thermal acclimation, amphipods were exposed to acutely declining oxygen tensions, where metabolic performance (MO_2_) and anaerobic metabolite concentration (lactate) were measured during the normoxic (80%–100% a.s.) and hypoxic phases (30% a.s.). The molecular mechanisms underpinning responses to these treatments were evaluated using RNA‐Seq.

## 2 MATERIALS AND METHODS

### 2.1 Physiological responses to hypoxia following thermal acclimation

Amphipods were collected from an intertidal mudflat at Saltash, UK (50°24'51.57'' N, 4°12'41.70'' W), and transported to the laboratory within 2 hr of collection. Individuals were acclimated to laboratory conditions for 7 days (*T* = 10°C, *S* = 32, 12hr‐L:12hr‐D, fed carrot *ad libitum*) prior to experimentation. Only adult males, identified using morphological criteria (Lincoln, 1979), were used in cross‐tolerance experiments (Figure A1 for experimental design) to minimize possible sex‐ and life cycle‐related effects. Individuals were acclimated for 7 days to one of two acclimation temperatures (*T_a_*) either 10°C (control) or 20°C using an experimental mesocosm system (2 experimental runs performed, Appendix S1 for mesocosm description). Temperatures were selected as they occur within the typical thermal range experienced in local estuaries (~3–20°C) (Uncles & Stephens, 2001). At the collection site, 10–20°C variations in temperature occur on a daily basis, and extremes of up to 32°C can occur in summer. The upper thermal tolerance of this population is ~35°C (Calosi et al., 2013); thus, 20°C constitutes a non‐lethal temperature suitable for longer term exposure. Following the acclimation period, individuals were abruptly transferred to a standardized test temperature (*T_t_*) of 10°C for determination of MO_2_. Amphipods in both acclimation treatments experienced similar levels of handling (see Appendix S1 for full details). This transfer method has been used in previous cross‐tolerance experiments of aquatic organisms (Todgham et al., 2005). Animals were allowed to rest for 1 hr following the temperature transfer similar to previous studies of temperature effects on *E. marinus* metabolism (Dorgelo, 1973). MO_2_ was determined using closed‐chamber respirometry (Appendix S1 for details). For each *T_a_*, 10 or 20ºC, individuals were allowed to deplete oxygen down to 80% a.s.in the normoxic control (10NO or 20NO) (over ~1 hr) and 30% a.s. in the hypoxic treatment (10HY or 20HY) (over ~5 hr). The normoxic controls were not overly influenced by handling as animals rapidly settled upon the mesh substratum within the respirometer and achieved a stable level of MO_2_ within 30 min. Once individuals had depleted the oxygen to these specified oxygen tensions, the respirometry chamber was opened, and the amphipod was removed immediately and blotted dry before the wet mass was measured using a microbalance (MSA225P‐000‐DA, Göttingen Sartorius AG, Germany, ±0.01 mg). Individuals were flash‐frozen in liquid nitrogen and stored at −80°C. MO_2_ was calculated for each individual (Appendix S1 and Figure A2). The first 30 min of data were removed to account for handling stress. Whole‐body lactate concentration was determined for frozen individuals using a commercial lactate assay kit (Lactate Kit 735‐10, Trinity Biotech, Ireland) similar to Collins et al. (2019) (Appendix S1 for details). In aquatic animals, reduced MO_2_ in response to hypoxia would typically indicate reduced performance. However, amphipods can regulate MO_2_ below the normoxic rate under declining oxygen, i.e. regulated suppression of MO_2_ by between 20% and 50% is possible (Sutcliffe, 1984; Verberk et al., 2018). Therefore, lactate concentration was measured as an indicator of anaerobic metabolism. Reduced MO_2_ accompanied by a transition to anaerobiosis under hypoxia would reflect reduced performance resulting from multiple stressors.

To test for differences in MO_2_ or lactate between treatments, two separate two‐way ANCOVAs were performed with acclimation temperature (10 or 20°C) and oxygen level (80% a.s. or 30% a.s.) as factors and mass as a covariate. MO_2_ and lactate data were first log‐transformed in order to meet assumptions of normality of residuals. A subset of individuals (*N* = 5 per treatment, wet mass = 87.3 ± 4.1 mg, mean ± *SEM*) was then used for RNA‐Seq analysis.

### 2.2 Transcriptomic responses to hypoxia following warm acclimation

To test the cellular mechanisms associated with altered hypoxic performance following warm acclimation, an RNA‐Seq experiment was performed using individuals exposed to the four treatments previously described.

### 2.3 Library preparation and RNA sequencing

Total RNA was isolated from individuals using the GeneJET RNA Purification Kit (Thermo Scientific, USA) (*N* = 5 per treatment). RNA integrity was assessed using Agilent Bioanalyzer 2100 (Agilent Technologies, USA). Library preparation and RNA sequencing were performed at the Beijing Genomics Institute, Hong Kong. A total of 20 TruSeq RNA libraries (Illumina, San Diego, USA) were prepared and sequenced on two lanes of an Illumina HiSeq 4000 sequencer using 100‐bp paired‐end sequencing (Illumina, San Diego, USA).

### 2.4 Gene expression analysis

Sequencing produced 431.2 M paired‐end reads (13.4–29.1 M paired‐end reads per sample). The sequenced reads were quality‐checked, assembled using Trinity v2.5.1 (Haas et al., 2013) and annotated using Trinotate v.3.2.0 (Appendix S1 for full description). Transcript expression was quantified using kallisto v0.44.0 (Bray et al., 2016) ran as a part of Trinity. Transcript expression was imported into R and summarized to the “gene” level using tximport v1.0.3 (Soneson et al., 2015). Differential expression analysis was performed using DESeq2 v1.12.4 (Love et al., 2014). Contigs with low counts (<5) were removed. Visual inspection of PCA plots of variance‐stabilized counts and sample distance matrices were utilized to remove outlier samples (Figure A3, one sample was removed from both 10NO and 10HY as they clustered separately along PC1 from the other four samples). Treatment (treatment: 10NO (*N* = 4),10HY (*N* = 4), 20NO (*N* = 5) and 20HY (*N* = 5)) and experimental run assigned as a batch variable (batch: one or two) were set as factors in the DESeq2 model (design = ~batch + treatment). DEGs (*p*_adj_ < .05) were identified from pairwise comparisons generated by DESeq2’s *results()* function which performs independent filtering of low counts and count outlier genes. Pairwise comparisons investigated were (a) changes to constitutive expression caused by warm acclimation under normoxic conditions (20NO vs. 10NO), (b) cold‐acclimated animals exposed to hypoxia compared to normoxia (10HY vs. 10NO) and (3) warm‐acclimated animals exposed to hypoxia compared to normoxia (20HY vs. 20NO). We tested for functional enrichment/overrepresentation of GO terms for the significantly differentially expressed genes (DEGs) (*p*_adj_ < .05, log_2_ fold change <−1 or >1) using GOSeq v1.24.0 (Young et al., 2010). “Gene” lengths are required to correct for annotation bias (longer genes more likely to be annotated). Transcript lengths were summarized to the “gene” level using the Trinity script “TPM_weighted_gene_length.py” and used only with GOSeq. GO terms with small numbers of transcripts were removed (≤5 transcripts) and were mapped to GO slim terms using GSEABase v1.34.1 (Morgan et al., 2016). To assess whether thermal acclimation altered the effect of hypoxia on global transcriptional profiles, PCA of batch‐corrected variance‐stabilized counts of all tested genes was generated via DESeq2 and limma v 3.28.21 (Ritchie et al., 2015) (*N* = 222,469 genes). A two‐way ANOVA followed by Tukey's post hoc test was performed on PCA scores with acclimation temperature and oxygen level as factors.

Frontloaded genes were identified based upon an adapted method of Barshis et al. (2013) which relies upon the results of the standard differential analysis conducted. To assess whether frontloading plays a role in stressor interactions, significant DEGs affected by hypoxic exposure in the cold‐acclimated hypoxia group were extracted (10HY vs. 10NO). Upregulated and downregulated DEGs were analysed separately. These genes were not significantly affected in the warm‐acclimated hypoxia group (20HY vs. 20NO). Two pieces of information were required to investigate frontloading: (1) raw fold change (“fold upregulation” =2^log_2_FC, “fold downregulation” =1/2^log_2_FC) and (2) normalized counts as a measure of constitutive expression between acclimation temperatures (batch‐corrected variance‐stabilized counts for 20NO and 10NO). We identified three main categories of response: (1) “Greater FC in warm acclimated”: genes which displayed a greater fold change in response to hypoxia in warm‐acclimated compared to cold‐acclimated group (raw FC 20HY vs. 20NO > raw FC 10HY vs. 10NO, ratio > 1); (2) “Frontloaded”: where a “reduced reaction” occurred in response to hypoxia in the warm‐acclimated compared to cold‐acclimated group as a result of greater constitutive expression resulting from warm acclimation under normoxic conditions (raw FC 20HY vs. 20NO < raw FC 10HY vs. 10NO, ratio < 1, counts 20NO > counts 10NO for upregulated DEGs or counts 20NO < 10NO for downregulated DEGs); and (3) “stress indicators” that also showed a “reduced reaction” to hypoxia in the warm‐acclimated group but lower constitutive expression following thermal acclimation under normoxia (raw FC 20HY vs. 20NO < raw FC 10HY vs. 10NO, ratio < 1, counts 20NO < counts 10NO for upregulated DEGs or counts 20NO > 10NO for downregulated DEGs) (Figure A4).

Finally, to investigate the association between frontloading and cross‐tolerance at the physiological level, we used a method of Veilleux et al. (2015). Variance‐stabilized counts of genes were correlated with MO_2_ using Pearson's correlation (with a significance threshold of *p* < .01). MO_2_ was first standardized (Veilleux et al., 2015) as the residuals from the regression line between mass and MO_2_ of the control group (10NO) (Figure A5). To also explore the broader relationship between global gene expression and performance, principal component scores of the whole transcriptome previously described were also correlated against standardized MO_2_.

## 3 RESULTS

### 3.1 Metabolic responses to hypoxia following warm acclimation

**FIGURE 1:**
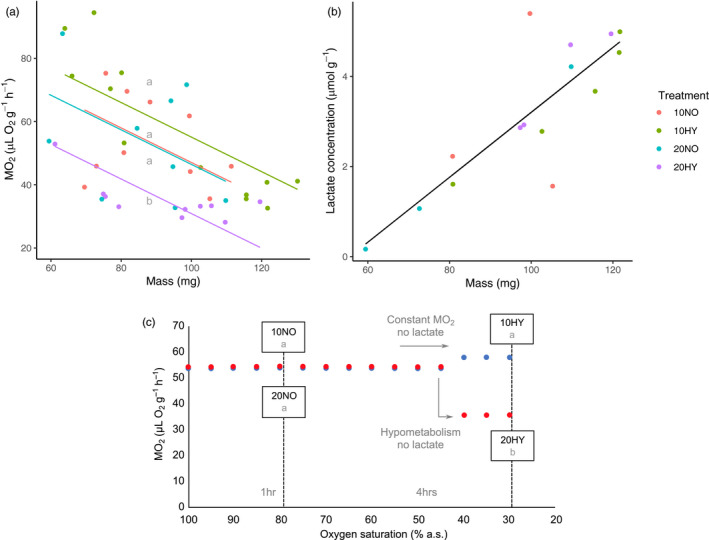
Physiological response of *Echinogammarus marinus* to hypoxia following thermal acclimation. (a) MO_2_ of *E. marinus* in response to temperature and hypoxia (10NO: *N* = 10; 20NO: *N* = 9; 10HY: *N* = 12; 20HY: *N* = 10). MO_2_ was similar between warm‐ and cold‐acclimated individuals under normoxia (20NO vs. 10NO). Under hypoxia, warm‐acclimated individuals displayed a significantly lower MO_2_ (*p* < .05) or hypometabolic shift not found in cold‐acclimated individuals (20HY vs. 10HY). Mass was a significant covariate (*p* < .05). (b) Lactate concentration was not significantly affected by any experimental treatment (*p* > .05) (10NO: *N* = 3; 20NO: *N* = 3; 10HY: *N* = 5; 20HY: *N* = 4). Line shown only for mass which was significantly related to lactate concentration (*p* < .05). (c) Sample utilization for transcriptomic analysis to investigate mechanisms underpinning performance (*N* = 5 whole individuals per treatment, Appendix S3 for sample list). Conceptual diagram redrawn from (a) and (b). We tested mechanisms elicited in response to hypoxia compared to normoxia between acclimation treatments (10HY vs. 10NO compared to 20HY vs. 20NO) and the effect of changes to constitutive gene expression during acclimation (20NO vs. 10NO). We were also interested in potential mechanisms associated with the hypometabolism displayed by warm‐acclimated individuals under hypoxia (20HY) that was not accompanied by increased anaerobiosis (lactate concentration)

The metabolic rate (MO_2_) of *E. marinus* was significantly affected by acclimation temperature (*F*_1,36_ = 13.35, *p* < .001), oxygen level (*F*_1,36_ = 4.28, *p* = .046) and their interaction (*F*_1,36_ = 7.78, *p* = .008) with mass as a significant covariate (*F*_1,36_ = 24.60, *p* < .001) (Figure 1a). *Post hoc* Tukey's tests did not detect significant differences (*p* > .05) in MO_2_ under normoxic conditions between acclimation temperatures or under hypoxia in cold‐acclimated individuals (10NO vs. 20NO vs. 10HY). However, MO_2_ under hypoxia was significantly lower in warm‐acclimated individuals compared to cold‐acclimated individuals (20HY vs. 10HY) (*p* < .001). This reduced level of MO_2_ in warm‐acclimated animals under hypoxia was also lower than under normoxic conditions (20HY vs. 20NO) (*p* = .011) which indicated that a hypometabolic shift had occurred as a result of warm acclimation. There were no significant effects of acclimation temperature or oxygen level on lactate concentration (*p* > .05) which was only significantly affected by mass (*F*_1,10_ = 22.3, *p* < .001) (Figure 1b, MO_2_ and lactate data, Appendix S2). Transcriptomic mechanisms associated with this hypometabolic response were investigated (Figure 1c).

### 3.2 Reduced transcriptomic response to hypoxia following warm acclimation

**FIGURE 2:**
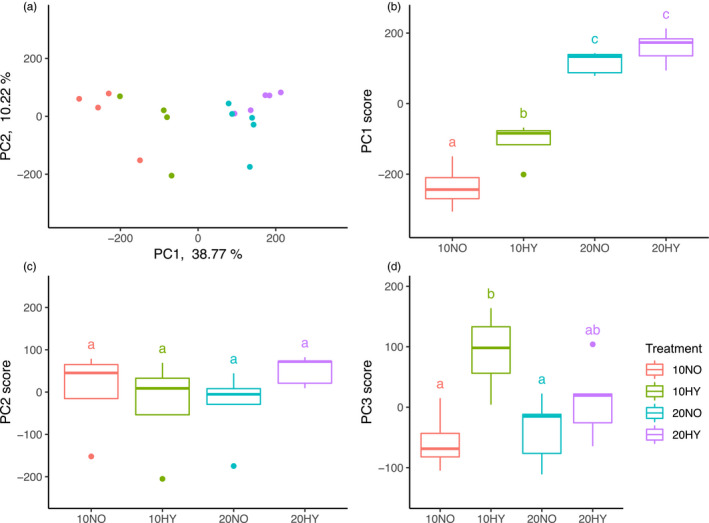
(a) PCA plot of all the genes in the transcriptome used for DE analysis (*N* = 222,469 genes, batch‐corrected variance‐stabilized counts) (B‐D) Box plot of scores for principle components 1–3 plotted against experimental treatment. PC1 scores differed significantly by temperature and oxygen. A greater difference in expression profiles was observed between normoxia and hypoxia in cold‐acclimated (10HY vs. 10NO) than warm‐acclimated (20HY vs. 20NO) animals. PC3 scores were significantly affected by oxygen only and again show greater differences for cold‐acclimated individuals (10HY vs. 10NO) than warm‐acclimated (20HY vs. 20NO). Letters indicate significant differences between treatments (*p* < .05)

The assembled transcriptome for *E. marinus* consisted of 383,395 transcripts corresponding to 252,460 genes (Trinity “genes”) (Figure A6 and Appendix S3 for assembly metrics). For all genes tested in the DE analysis (*N* = 222,469 genes), samples were separated by treatment along PC1 accounting for 38.77% of the variance (Figure 2a, for further biplots of principal components, see Figure A7). The top 100 genes contributing to this component were significantly enriched for GO terms related to polysaccharide metabolism and extracellular region. PC1 scores were significantly affected by temperature (*F*_1,14_ = 164.18, *p* < .001) and oxygen (*F*_1,14_ = 12.28, *p* = .004) with greatest separation observed between different acclimation temperatures (Figure 2b). Temperature drove the largest change in expression profiles with significantly altered expression of 27,366 genes elicited under normoxic conditions (20NO vs. 10NO, 3,941 upregulated, 23,425 downregulated, Figure A8). The changes included widespread enrichment of GO terms associated with metabolism, protein turnover and ion transport. Metabolic reorganization may be indicated by upregulation of oxygen transport genes by respiratory pigments such as haemocyanin, but downregulation of aerobic metabolic genes. The latter included downregulation of TCA cycle genes, such as citrate synthase, and mitochondrial electron transport chain genes, such as cytochrome c oxidase. Changes to protein turnover included downregulation of genes enriched for GO terms involved in protein synthesis and unfolded protein binding consisting primarily of downregulated heat‐shock protein genes.

PC2 was not significantly affected by treatment (Figure 2c). Separation was also observed along PC3, accounting for 7.61% of the variation (Figure A7), and the top 100 genes contributing to this component were significantly overrepresented for GO terms associated with cuticle structure and chitin metabolism. PC3 scores were significantly affected by oxygen only (*F*_1,14_ = 12.22, *p* = .004) (Figure 2d). Along both PC1 and PC3, warm‐acclimated individuals differed less in expression profiles between normoxic and hypoxic conditions (20NO vs. 20HY) compared to cold‐acclimated individuals (10NO vs. 10HY). PC1 and PC3 scores were only significantly different in the cold‐acclimated hypoxic exposures (10HY vs. 10NO, PC1: *p* = .017, PC3: *p* = .016) and not the warm‐acclimated group (20HY vs. 20NO, *p* > .05) (Figure 2b and d). Tests of correlation between MO_2_ and principal component scores found that none of the principal components were significantly correlated with MO_2_ (*p* > .05).

Reduced changes to expression under hypoxia may also be indicated by the 91.5% reduction in the total number of genes significantly regulated by hypoxia in the warm‐acclimated (20HY vs. 20NO) compared to the cold‐acclimated group (10HY vs. 10NO). In the cold‐acclimated group (10°C), hypoxia elicited significant changes in the expression of 1,033 genes (10HY vs. 10NO, 318 upregulated, 715 downregulated, Figure A8). Upregulated DEGs were significantly enriched for GO terms linked to cytoskeleton, protein binding and immune responses. Downregulated DEGs were enriched for GO terms linked to protein synthesis and translation. For warm‐acclimated individuals (20°C), hypoxia significantly affected the expression of only 88 DEGs (20HY vs. 20NO, 22 upregulated, 66 downregulated, Figure A8). No significant functional enrichment of genes was observed for either upregulated or downregulated DEGs. Upregulation of a small number of heat‐shock protein genes was observed (Appendix S3 and Appendix S4 for differential expression, PCA and GO enrichment data).

### 3.3 Transcriptional frontloading may be associated with a reduced transcriptional response between stressors

**FIGURE 3:**
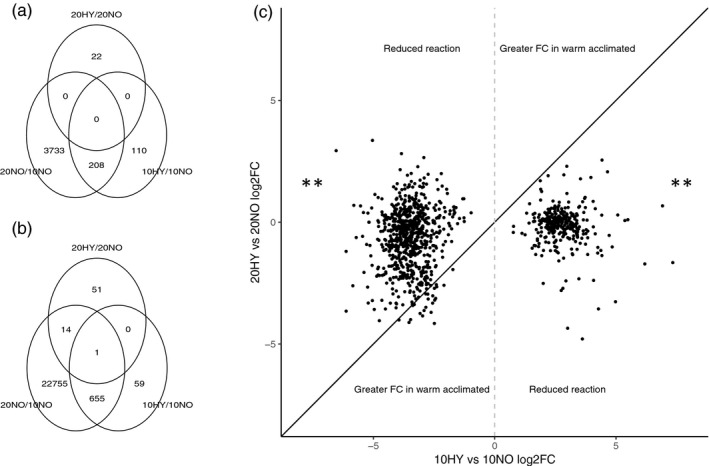
Relationship between changes to gene expression in response to hypoxia between warm‐ and cold‐acclimated treatments. (a) Venn diagram of upregulated DEGs between treatments including changes resulting from thermal acclimation (20NO/10NO) and hypoxic exposure within the cold‐ (10HY/10NO) and warm‐acclimated individuals (20HY/20NO) and similarly for (b) showing downregulated DEGs. (c) For all 1,030 unique genes with expression significantly affected (*p*_adj_ < .05 by hypoxia in cold‐acclimated animals only (genes with no overlap with 20HY/20NO in Venn diagrams), log_2_ fold changes between normoxic and hypoxic animals are shown for cold‐acclimated (10HY vs. 10NO, *x*‐axis) against warm‐acclimated individuals (20HY vs. 20NO, *y*‐axis). If the log_2_ fold changes are equal, points will fall upon the line of equality (solid black line); thus, hypoxia affects expression in a similar manner in both acclimation temperatures. For upregulated DEGS (log_2_FC > 0, right of grey dashed line), points above the line of equality indicate a greater magnitude of response to hypoxia in warm‐acclimated individuals. Points below the line indicate a “reduced reaction” in warm‐acclimated individuals. Asterisks indicate that a significant deviation away from a 50:50 distribution of log_2_ fold changes around a line of equality was observed in a chi‐squared test. Upregulated (*n* = 317, *X*_2_ = 317, *p* < 2.2e−16) and downregulated DEGs (*n* = 713, *X*_2_ = 623.97, *p* < 2.2e−16) were analysed separately

A greater difference in expression profiles was observed between normoxia and hypoxia in cold‐acclimated (10HY vs. 10NO) than warm‐acclimated (20HY vs. 20NO) animals which may be associated with frontloading. Responses to hypoxia were unique for each acclimation temperature with very little overlap observed between DEGs (only 1 gene in common between 10HY vs. 10NO, compared to 20HY vs. 20NO, Figure 3a and b). 1,030 DEGs unique to the cold‐acclimated hypoxia group (10HY vs. 10NO) were explored for frontloading. Of the 317 unique upregulated DEGs, all were “reduced reaction” genes (Figure 3c). For the 713 unique downregulated DEGs, 690 were also “reduced reaction” genes with only 23 showing a greater fold change under warm acclimation (Figure 3c).

**FIGURE 4:**
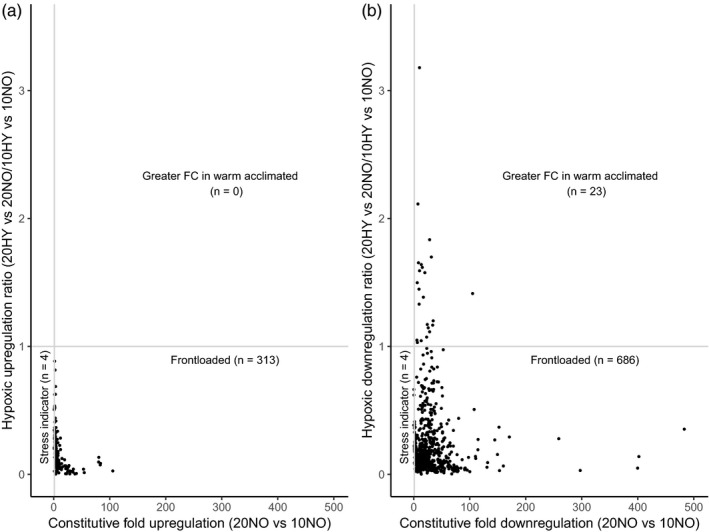
(a) Raw fold changes in constitutive expression between warm‐acclimated and cold‐acclimated animals under normoxia (20NO vs. 10NO, on the *x*‐axis) against the ratio of raw fold upregulation of genes (*n* = 317) between warm‐acclimated and cold‐acclimated animals under an acute hypoxic exposure (ratio of 20HY vs. 20NO: 10HY vs. 10NO, on the *y*‐axis) (b) Raw fold changes in constitutive expression between warm‐acclimated and cold‐acclimated animals under normoxia (20NO vs. 10NO, on the x‐axis) against the ratio of raw fold downregulation of genes (*n = *713) between warm‐acclimated and cold‐acclimated animals under an acute hypoxic exposure (ratio of 20HY vs. 20NO: 10HY vs. 10NO, on the y‐axis). Ratios > 1 on the y‐axis (grey horizontal line) indicate a greater reaction to hypoxia in the warm‐acclimated group (20HY vs. 20NO fold up/downregulation > 10HY vs. 10NO fold up/downregulation). Ratios < 1 indicate a “reduced reaction” to hypoxia in the warm‐acclimated individuals (20HY vs. 20NO fold up/downregulation < 10HY vs. 10NO fold up/downregulation) which can be further subdivided into “frontloaded” genes with greater constitutive expression (*x*‐axis > 1, grey vertical line) or “stress indicator” genes with lower constitutive expression (*x*‐axis < 1)

98.7% of the 317 upregulated DEGs were frontloaded, and the remaining four were stress indicator genes (Figure 4a). Frontloading was also the predominant response observed for the 713 downregulated DEGs, with a total of 686 frontloaded genes (96.2%). The remaining genes consisted of four stress indicator genes and 23 genes with greater fold change in the warm‐acclimated group (Figure 4b). Only the frontloaded genes showed any significant GO enrichment for both upregulated and downregulated DEGs. The upregulated frontloaded transcripts consisted of GO terms associated with cytoskeleton, protein binding and immune response (Figure A9). The downregulated frontloaded genes were significantly enriched for multiple GO terms involved in protein synthesis (e.g. ribosomal protein subunits), translation and RNA binding (Figure A10, Appendices S3 and S4 for frontloaded gene and GO enrichment data). Of the frontloaded DEGs, none were significantly correlated with whole‐organism MO_2_ (*p* > .01).

## 4 DISCUSSION

Understanding biological mechanisms underpinning stressor interactions is key to predicting the consequences of rapid environmental change (Gunderson et al., 2016; Kelly et al., 2016). This study provides evidence, for the first time, that transcriptional frontloading contributes to cross‐tolerance between stressors. Acclimation to one stressor (temperature) altered molecular responses and physiological performance under a second stressor (hypoxia) in an ecologically important marine invertebrate. Warm acclimation prior to hypoxic exposure led to constitutive expression of multiple genes that are important in the reaction to hypoxia, predominantly those involved in mechanisms associated with the stress response and reduction of energetically costly cellular processes. We therefore propose transcriptional frontloading as a key mechanism by which organisms face the challenge of multiple stressors.

### 4.1 Warm acclimation altered physiological responses to hypoxia

Interactions between stressors upon the same physiological trait create the possibility of interactions at lower levels of organization (Todgham & Stillman, 2013). For temperature and hypoxia, interactions occur through effects on aerobic metabolism (Pörtner, 2010). In *E. marinus*, warm acclimation resulted in a hypometabolic response under hypoxia, which was not observed in cold‐acclimated individuals. Reduced MO_2_ may arise as a result of either downregulation of energy turnover or decreased activity (Speers‐Roesch et al., 2018). In this study, activity was minimized for all individuals by the small size of respirometers and provision of substratum to cling to, mimicking the small crevices beneath rocks and algal thalli, in which this species settles upon in the wild. The response of *E. marinus* is therefore likely to represent reduced energetic demand. Interpretation of whether reduction in MO_2_ alone is beneficial in response to environmental drivers is somewhat subjective (Seebacher et al., 2015). However, this hypometabolic response was not accompanied by an increase in lactate, the primary anaerobic end product for crustaceans (Harrison, 2015). Thus, this does not reflect a loss of capacity to regulate aerobic metabolism as it was not accompanied by anaerobiosis associated with time‐limited survival (Boutilier & St‐Pierre, 2000; Grieshaber et al., 1994). This reduction in MO_2_ may instead reflect an adaptive strategy involving regulated hypometabolism, which can be common in gammarid amphipods and is thought to contribute to hypoxic performance (Verberk et al., 2018), and may be an important strategy to weather short episodes of hypoxia typical of estuaries until favourable conditions return (Larade & Storey, 2002; Tyler et al., 2009). We therefore suggest that the transcriptional changes observed are consistent with the notion of a regulated hypometabolism and maintenance of performance.

### 4.2 Transcriptional frontloading operates between temperature and hypoxia at the molecular level

The underpinning cellular mechanisms which generate cross‐tolerance between temperature and hypoxia remain largely unknown (Todgham et al., 2005). From single stressor studies, transcriptional frontloading can induce a heightened ability to deal with acute stress which may be represented at the transcriptional level by a reduction in the number of significantly affected genes (i.e. due to changes to constitutive gene expression, less of an inducible stress response may be required to cope with a stressor) (Barshis et al., 2013). Warm‐acclimated *E. marinus* displayed reduced changes to global gene expression profiles under hypoxia compared to cold‐acclimated individuals which may indicate reduced sensitivity. The more limited response may be associated with transcriptional frontloading which was the predominant mechanism occurring between these stressors with ~96%–98% of all significant DEGs being classified as frontloaded. Thus, those genes significantly affected by hypoxia in the cold‐acclimated group were not significantly affected in the warm‐acclimated group, which may be related to greater constitutive expression.

From previous studies, we predicted cellular defences to be amongst the upregulated frontloaded genes (Barshis et al., 2013; Clark et al., 2018; Kenkel et al., 2013). We observed a complex stress response for this species. Heat‐shock protein genes (HSPs) did not appear to be frontloaded, which could reflect the nature of the acute stressor (hypoxia and not acute heat shock) (Barshis et al., 2013). Although, from the differential expression analysis, a small number of HSPs were upregulated in the warm‐acclimated but not cold‐acclimated hypoxia group. This could reflect cellular stress or, alternatively, could be associated with hypometabolism, as HSP upregulation has been considered a preparative change to preserve the proteome whilst in a metabolically depressed state (Storey & Storey, 2011).

Frontloading of genes involved in several other stress pathways was observed. Upregulated frontloaded genes were widely enriched for immune responses similar to corals (Barshis et al., 2013), which may reflect a protective strategy. Also, cytoskeleton‐ and microtubule‐related processes were enriched for frontloaded genes and have been implicated in the responses of intertidal invertebrates to stress (Clark et al., 2018). Thus, inability to detect significant upregulation in the warm‐acclimated hypoxia group could indicate reduced levels of stress and potentially lower maintenance costs under hypoxia.

Frontloading was also prevalent within the downregulated genes. The adaptive value of downregulated frontloaded genes in response to temperature alone has not been elucidated but has been suggested to include transcription factors with potential consequences for expression of other genes (Barshis et al., 2013). In the cold‐acclimated group, the hypoxic response consisted mainly of downregulated ribosomal pathways, translation and RNA binding, suggesting a reduction in costly protein synthesis in common with other marine species exposed to hypoxia (Gracey et al., 2001). In comparison, neither ribosomal pathways nor any other biological processes were significantly enriched upon hypoxic exposure in the warm‐acclimated group, which may be attributable to frontloading of these groups of genes during acclimation. Hypoxia‐related changes to protein synthesis gene expression have previously been found to be modifiable by other environmental factors in crustaceans, but only for hypercapnia (Rathburn et al., 2013). Changes to protein synthesis have, however, received attention in response to temperature stress in isolation in gammarids where reduced protein synthesis rates may conserve energy in highly variable environments (Rastrick & Whiteley, 2013).

The cellular interaction between these stressors is dominated by downregulation of costly cellular processes, which seems consistent with the notion of hypometabolism at the organismal level. Previous frontloading studies have iterated a need to identify genes underpinning physiological performance (Barshis et al., 2013). Whilst there is clearly a protective effect of frontloading at the cellular level, frontloaded genes did not correlate strongly with the hypometabolic response of *E. marinus*. Elucidating how gene expression scales to physiological performance in non‐model marine invertebrates under hypoxia remains challenging (Spicer, 2014), as noted in other non‐model organisms (Todgham et al., 2005). Particularly for crustaceans, understanding gene functionality may be hindered by low annotation rates (Das et al., 2016).

Difficulties in linking gene expression and MO_2_ could also reflect that transcriptional responses were measured at a single time point. Hypometabolism is subject to considerable modification at the protein level (e.g. protein phosphorylation) (Storey & Storey, 2004). The widespread enrichment of frontloaded genes for translation and protein synthesis pathways may suggest regulation at the proteome level. Further work could aim to identify post‐translational modifications or rates of protein synthesis associated with the putative function of these frontloaded genes. Time course and gene regulatory network analyses (Sleight et al., 2020) may also help to elucidate the complex relationships between frontloaded genes and cross‐tolerance.

Physiological and molecular responses could also potentially be affected by methodological factors. A potential challenge associated with any cross‐tolerance or frontloading study is the requirement for standardized test temperatures to directly identify improved/impaired performance between animals with different thermal histories. Here, we abruptly transferred warm‐ and cold‐acclimated animals to the cold acclimation temperature similar to a previous cross‐tolerance study on intertidal fish (Todgham et al., 2005). Potentially, this transfer could be predicted to have a greater effect on the responses of warm‐acclimated individuals. However, the cold test temperature of 10ºC represents a common habitat temperature well tolerated by this species and not an environmental extreme. Additionally, a temperature reduction of 10ºC falls within the normal daily variation experienced by this species. Our characterization of metabolic and transcriptomic responses within the same individual meant that there was some variation in time spent within the respirometer. However, we opted to standardize by end PO_2_ in the respirometer rather than time as transcriptomic responses of estuarine amphipods can differ markedly dependent upon hypoxic severity (Collins et al., 2019). It is unlikely that different lengths of time spent in the respirometer between the normoxic and hypoxic treatments overly influenced the outcomes. *E. marinus* rapidly recovered from handling and achieved a stable MO_2_ within 30 min after introduction into the respirometer, similar to other gammarid amphipods (Hervant et al., 1999). Furthermore, MO_2_ was not raised under normoxia compared to hypoxia in cold‐acclimated animals (10HY vs. 10NO). If handling stress was occurring, MO_2_ would be consistently higher under normoxia compared to hypoxia for both temperature treatments, which was not the case. This suggests the observed responses were attributable to temperature effects.

In conclusion, transcriptional frontloading during thermal acclimation can prime the transcriptome for hypoxia and enable individuals to maintain performance through hypometabolism. The presence of transcriptional frontloading could reflect an adaptive, protective response at the cellular level to deal with multiple stressors. Frontloading has a complex role in cross‐tolerance and may enable some organisms to better persist in dynamic environments increasingly challenged by multiple anthropogenic threats.

## CONFLICT OF INTEREST

None declared.

## AUTHOR CONTRIBUTIONS

MC, JIS and MT conceived the study. MC performed experiments. MC, JIS, MSC and MT analysed data. All authors contributed to writing of the manuscript.

## Supporting information

Appendix S1

Appendix S2

Appendix S3

Appendix S4

## Data Availability

Data for this study is available from the supplementary information. Sequenced data are publicly available from the European Nucleotide Archive (BioProject: PRJEB34316).
