# Supplementary material for: Transcriptional frontloading contributes to cross‐tolerance between stressors
Source: Evol Appl. 2020 Oct 22;14(2):577–87. doi: 10.1111/eva.13142 (PMC7896706; doi:10.1111/eva.13142)
Supplement: Supplementary file 1 — Appendix S1 [file EVA-14-577-s001.pdf]

Supplementary Methods

Testing for cross-tolerance between temperature acclimation and hypoxia

In order to test for cross-tolerance between thermal acclimation and hypoxia, we utilised a similar approach to Todgham *et al.*, (2005), but focusing on longer term thermal acclimation rather than acute. Animals were acclimated to cold (10 °C) or warm (20 °C) temperature for 1 week, before hypoxic responses were tested at a standardised test temperature of 10 °C post-acclimation (Fig. A1). A standardised test temperature is essential to facilitate direct identification of improved/impaired performance between warm and cold acclimated animals (Huey and Berrigan, 1996). Transfer to a standard test temperature is also appropriate for application to the investigation of frontloading, as previous studies have taken animals from thermally different environments and tested responses (albeit thermal performance) under standard test conditions (Barshis *et al.*, 2013). We opted to test hypoxic responses of animals at a standardised cold/control temperature (10 °C in our case) similar to previous cross-tolerance studies (Todgham *et al.*, 2005). The experimental design is given in Fig. A1.

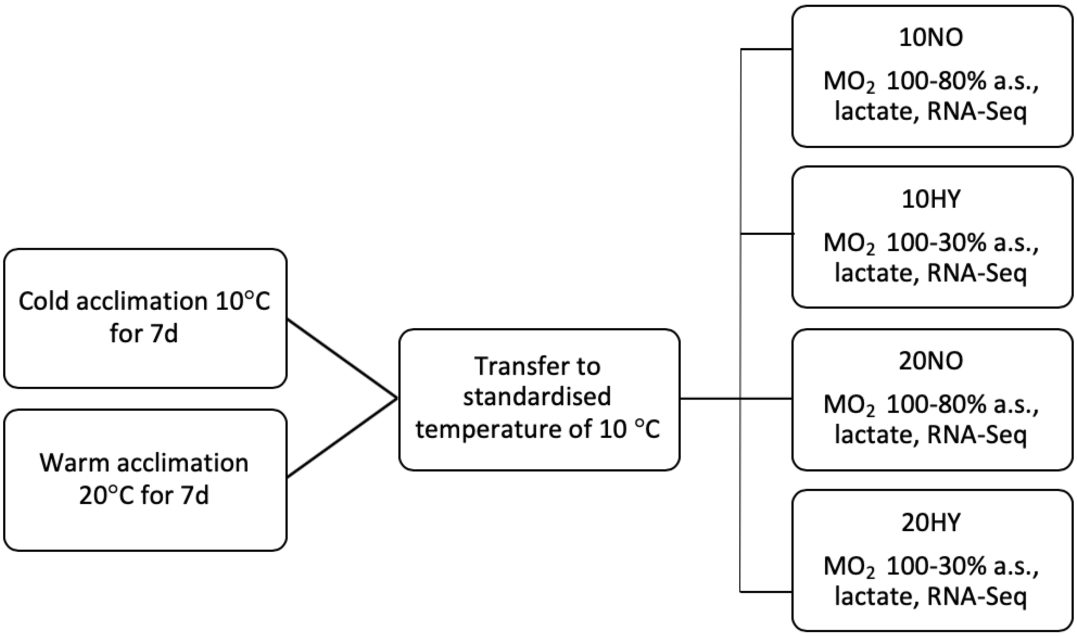

Fig. A1 Experimental design for cross-tolerance experiment. Animals were acclimated to different temperatures before being abruptly transferred to a standardised test temperature for determination of hypoxic responses. Individuals were allowed to settle for 1h before MO_2 was then measured under declining oxygen tensions using closed chamber respirometry. Individuals were allowed to deplete oxygen down to 30% a.s. for the hypoxic treatments for 10 °C acclimated (10HY) or 20 °C acclimated animals (20HY). Normoxic controls were used for each acclimation temperature (10NO and 20NO) where MO_2 was measured from 100 to 80% a.s. Individuals were frozen once they had reached the specified oxygen tension for lactate concentration analysis or RNA-Seq.

Acclimation to cold or warm temperature for 1 week was achieved using a mesocosm system. Amphipods were kept in sealed aquaria (vol. = 1.4 L) (*N* = 6 per treatment, 2 - 5 individuals in each, 2 experimental runs) each partially immersed in plastic trays filled with deionised water at the appropriate acclimation temperature. Water for the control temperature was reduced to T = 10 °C (9.9 ± 0.24) using a water chiller (L-350 Water Chiller, Guangdong Boyu Group Co.,Ltd., China) and that for the higher temperature of T = 20 °C (20.9 ± 0.3) was supplied with a water heater (100 W aquarium heater, EHEIM GmbH & Co KG, Germany). Water was circulated

around the tray using a pump (Koralia Nano Evolution 900 Circulation Pump, Hydor, Italy). Each aquarium was aerated by an air stone connected to an air pump (Mistral 2000, Aqua Medic GmbH, Germany) and air was scrubbed for carbon dioxide to minimise hypercapnia by passing it through a trap filled with NaOH (0.25 -1 mol L^{-1}, Sigma-Aldrich, Germany). Complete water changes were carried out every 3 - 4 d. Other environmental factors were kept constant (O_2 = 91.0 ± 1.7 % a.s., S = 32.0 ± 1.5, pH = 8.1 ± 0.15). Water temperature and oxygen partial pressure were measured daily using an oxygen microsensor (Pm-Pst7, Presens, Germany) and temperature probe (Pst 100, Presens, Germany) with a dissolved oxygen meter (Microx 4, Presens, Germany). Salinity and pH were measured every 1 - 2 d using a hand-held refractometer (HI96822 Digital Refractometer, Hanna Instruments, USA) and pH meter (S400 SevenExcellence pH/mV meter, Mettler-Toledo International Inc., USA) respectively. Following the acclimation period, animals were transferred from the mesocosm system to a thermostatically controlled temperature room at 10 °C. An abrupt transfer was used similar to Todgham *et al.*, (2005).

<u>Physiological and biochemical responses to hypoxia following thermal acclimation</u>

<u>MO_2 determination and analysis</u>

Individuals were allowed to settle for 1h in the controlled temperature room prior to MO_2 determination. Animals had been starved for at least 12h prior to measurement of MO_2 to minimise any contribution to MO_2 from digestion. Individuals were carefully transferred to a respirometer (vol. = 5 mL) and sealed. The respirometer contained an oxygen sensor spot (Presens, Germany) and magnetic flea to ensure adequate mixing within the chamber. The amphipods were separated from the magnetic flea by mesh (size = 2 mm) which also acted as substrate for the amphipod. Oxygen

levels within the chamber was measured at 10 min intervals using a fibre-optic cable and Fibox 4 meter (Presens, Germany). Individuals were allowed to deplete oxygen down to 80% a.s. and treated as normoxic controls for each acclimation temperature (T_a = 10 or 20 °C) (10NO or 20NO). For calculation of MO_2, the first 30 mins of data were removed to account for handling stress. For normoxic individuals, MO_2 was calculated for each individual by fitting a straight line between oxygen saturation and time (lm() function). The predicted values were used to calculate MO_2. In hypoxic individuals allowed to deplete oxygen down to 30% a.s. (10HY or 20HY), MO_2 may not necessarily be maintained at 100% of the normoxic rate in amphipods (Verberk *et al.*, 2018). To allow for this possibility, a segmented regression was fitted between oxygen saturation and time (Spicer and El-Gamal, 1999) using the package segmented v 0.5-3.0 (Muggeo, 2008). For hypoxic individuals, different rates of regulated MO_2 (termed R_1MO_2 and R_2MO_2) were identified for each individual (Fig. A2). R_2MO_2 was used to represent MO_2 of hypoxic individuals in subsequent analyses, as it was representative of their MO_2 at the time they were sampled.

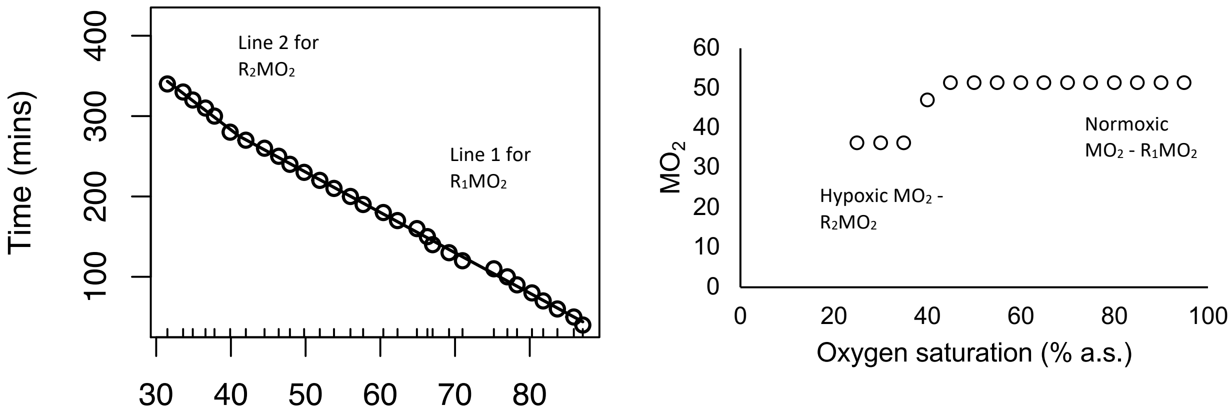

Fig. A2 Raw data showing time versus PO_2 for an individual. Oxygen decreases in a closed respirometry chamber through an organism's oxygen consumption. A subtle reduction in the rate at which oxygen decreases within the chamber occurs at approximately 40% a.s. for this individual. Therefore a segmented regression was fitted using the segmented package (Muggeo, 2008). Predicted values were extracted from the line at 5% a.s. intervals using segmented's broken.line() function and used to calculate MO_2 at different levels of oxygenation from 100% down to 30% a.s.

Lactate concentration

Frozen individuals were manually homogenised on ice in 500 µL of 10% TCA (Fisher Scientific, UK) and centrifuged for 3 min at 13000 rpm and 4 °C. The concentration of L-lactate was quantified using a lactate assay kit (Lactate Kit 735-10, Trinity Biotech, Ireland). 10 µL of the supernatant was added to 100 µL lactate reagent and incubated at room temperature for 15 min. Absorbance (λ = 540 nm) of this mixture was measured using a microplate reader (Versamax Microplate Reader, Molecular Devices LLC, California, USA) and calibrated against lactate standards.

Bioinformatic analyses

Transcriptome assembly

Sequencing produced 431.2 M paired-end reads. Adapter-trimming and removal of low-quality reads was performed by the sequencing facility (PHRED score < 7 across > 40 % of bases). Reads were quality checked using FastQC v0.11.5 (Babraham Bioinformatics). To verify there were no extraneous or low quality sequences remaining, reads were trimmed using Trimmomatic v0.32 (Bolger *et al.*, 2014) with the default Trinity parameters (SLIDING WINDOW 4:5, LEADING: 5, TRAILING: 5, MINLEN: 25). Reads were assembled using Trinity v 2.5.1 (Haas *et al.*, 2013) with all parameters set to default except the flag –min_kmer_cov = 2 due to memory requirements.

Assembly quality assessment

To assess quality of the assembled transcriptome, basic statistics and the ExN50 statistic were generated using utility scripts provided by Trinity (TrinityStats.pl and contig_Ex_N50_statistic.pl). The proportion of reads mapping against the assembled transcriptome was assessed using Bowtie2 v2.3.3.1 (Langmead and Salzberg, 2012) and samtools v1.7 (Li *et al.*, 2009). Transcriptome completeness was assessed using BUSCO v3.1.0 (Waterhouse *et al.*, 2017) by comparing the assembled transcriptome against the arthropod ortholog dataset (arthropoda odb9). The de novo assembly was annotated using Trinotate v 3.2.0 (trinotate.github.io) using default parameters. First, predicted protein-coding regions were generated from the transcripts using Transdecoder v 5.0.2. The transcripts and the translated protein sequences were searched, *via* Blastx/Blastp from Blast+ v2.7.1, against the Uniprot/Swissprot database provided by Trinotate (uniprot_sprot.pep file) with an e-

value cutoff of $1x10^{-5}$. Translated proteins were also searched using Hmmer v3.1b2 against the PFAM database provided by Trinotate (Pfam-A.hmm file). GO annotations were derived using Trinotate from Uniprot and PFAM mappings to GO terms.

Exploratory gene level analyses

Exploratory PCA analysis of variance stabilised counts was conducted prior to differential expression analyses using prcomp() to explore sources of variation in the global transcriptional profiles. Genes with low counts (<5) were first removed. For the cold acclimated treatments, two outlier samples were removed (10HY3 and 10NO4) as they clustered separately from the other eight samples along PC1 and in sample distance matrices (Fig. A3). Separation along PC1 (~33% variance) was driven by treatment but a batch effect of experimental run was observed along PC2 (~10% variance) (Fig. A3) and therefore batch was included as a covariate (~batch+treatment) for the DESeq2 model.

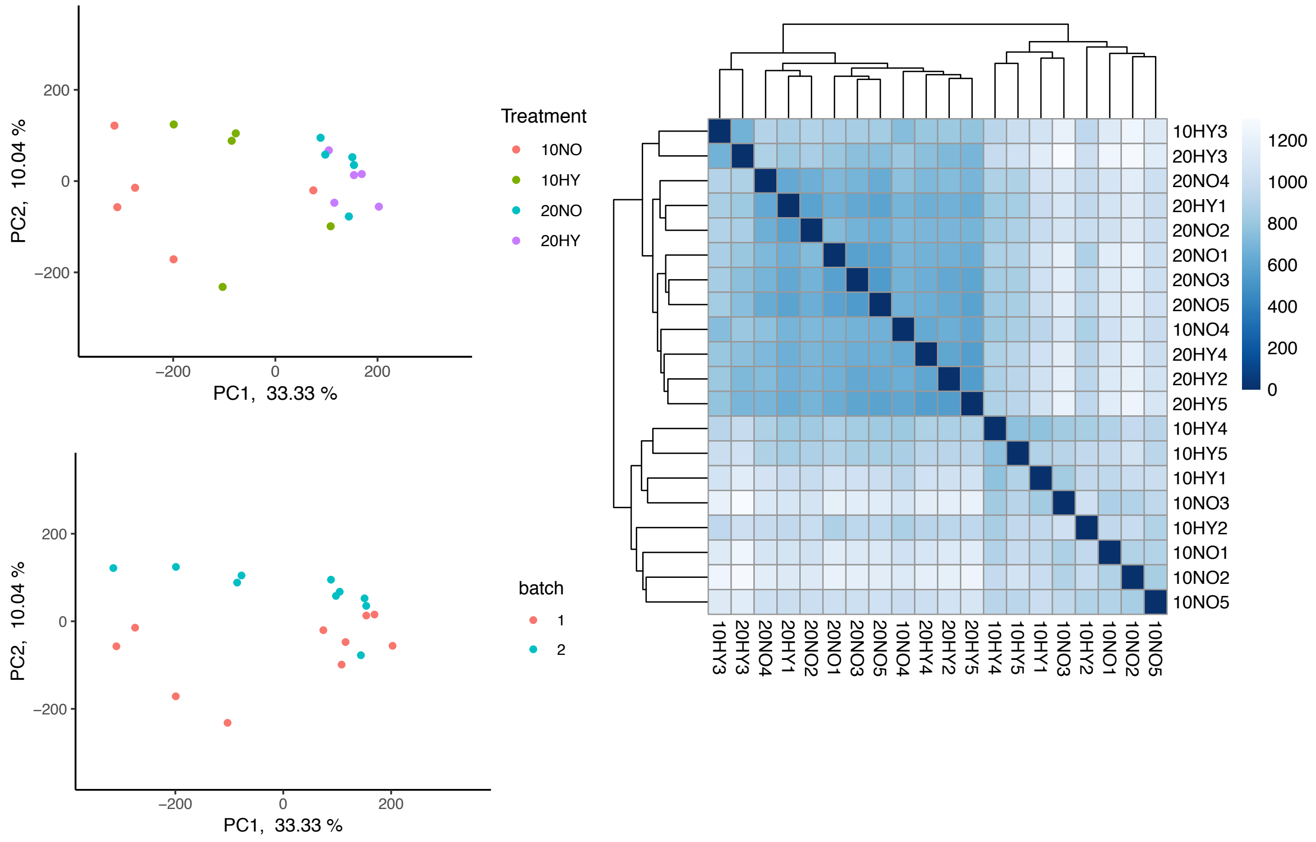

Fig. A3 Exploratory analysis of counts data. Two outlier samples (10HY3 and 10NO4) were identified from PCA plot of variance stabilised counts and sample distance matrix of variance stabilised counts. PCA plot of variance stabilised counts showed a batch effect along PC2 (10.04% of variance).

Identification of frontloaded genes

Frontloaded genes were identified following the framework presented in Barshis *et al.*, (2013). Building on this study, we investigated the consequences of changes to constitutive expression resulting from thermal acclimation under normoxic conditions (20NO vs 10NO) for the reaction to acute hypoxic stress (FC 20HY vs 20NO compared to 10HY vs 10NO). Criteria utilised to identify frontloaded genes is provided (Fig. A4).

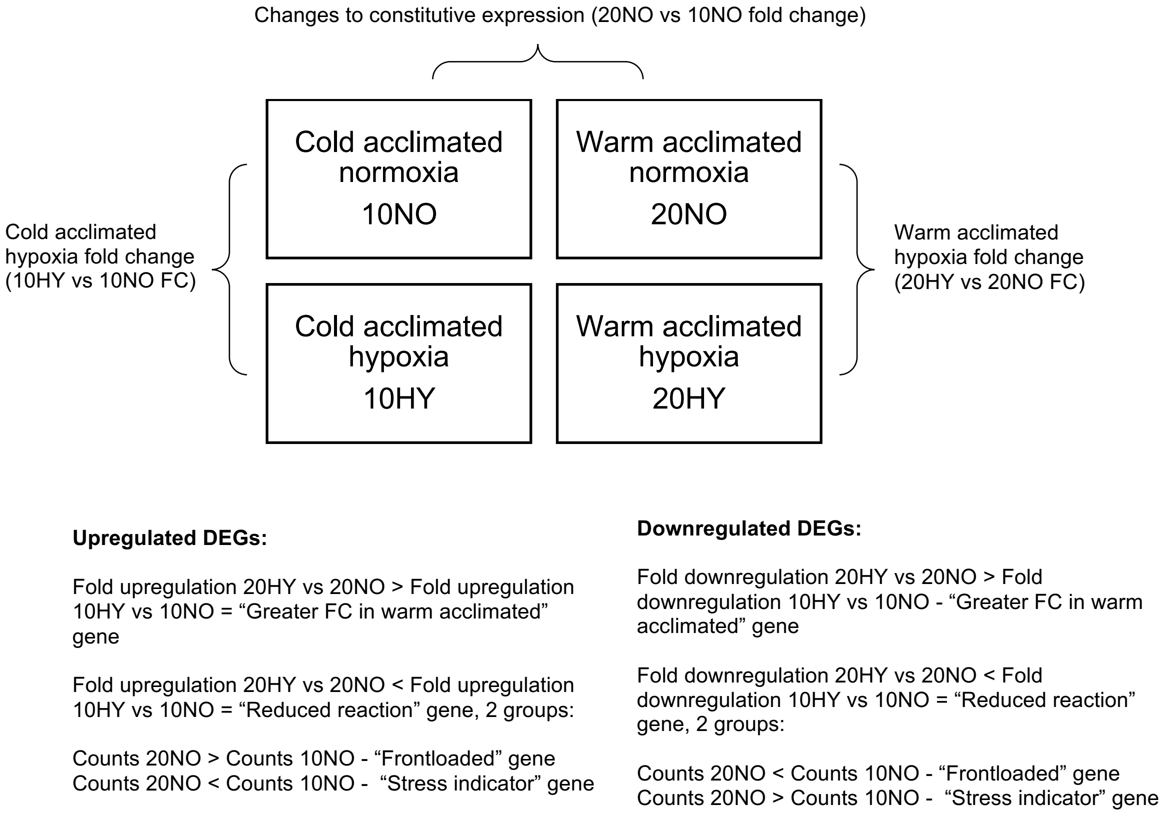

Fig. A4 Experimental design for frontloading. In the current study, changes to constitutive expression following warm acclimation (20NO vs 10NO) may have consequences for the magnitude of reaction to acute hypoxic stress (FC 20HY vs 20NO: FC 10HY vs 10NO). Criteria used to separate DEGs into response categories in the current study. We characterised 3 groups of genes: “Greater FC in warm acclimated”, “frontloaded” and “stress indicator” genes.

To assess whether frontloading was occurring, significant DEGs for the cold acclimated group in response to hypoxia (10HY vs 10NO, $P_{adj} < 0.05$) were extracted and filtered for those genes that were “unique” and not significantly affected by hypoxia in the warm acclimated group (20HY vs 20NO). From venn diagrams (Fig. 3 of main manuscript), “unique” genes included those contained within the sections 10HY vs 10NO and overlap of 10HY vs 10NO and 20NO vs 10NO, but not genes in 10HY vs 10NO which showed any overlap with 20HY vs 20NO. Upregulated and downregulated DEGs were analysed separately. Two pieces of information were

used to investigate frontloading: (1) raw fold change (2^log2FC), we considered “fold upregulation” (2^log2FC) for upregulated DEGs and for downregulated DEGs “fold downregulation” (1/2^log2FC). Log2FC were extracted from DESeq2 tables produced by results() function for the pairwise comparisons of interest (2) normalised counts as a measure of constitutive expression between acclimation temperatures. (batch-corrected using limma v 3.28.21 removeBatchEffect() (Ritchie *et al.*, 2015) on variance stabilised counts for 20NO and 10NO, also visually checked that counts match direction of fold change from results()). We categorised genes into three main types of response similar to Barshis *et al.*, (2013): (1) “Greater FC in warm acclimated”: genes which displayed a greater fold change in response to hypoxia in warm acclimated compared to cold acclimated group (raw FC 20HY vs 20NO > raw FC 10HY vs 10NO, ratio > 1), (2) “Frontloaded”: where a “reduced reaction” occurred in response to hypoxia in the warm acclimated compared to cold acclimated group as a result of greater constitutive expression resulting from warm acclimation under normoxic conditions (raw FC 20HY vs 20NO < raw FC 10HY vs 10NO, ratio < 1, counts 20NO > counts 10NO for upregulated DEGs or counts 20NO < 10NO for downregulated DEGs) and (3) “stress indicator” genes that also showed a reduced reaction to hypoxia in the warm acclimated group but showed lower constitutive expression following thermal acclimation under normoxia (raw FC 20HY vs 20NO < raw FC 10HY vs 10NO, ratio < 1, counts 20NO < counts 10NO for upregulated DEGs or counts 20NO > 10NO for downregulated DEGs). Some genes may display different directions of fold change in 10HY vs 10NO compared to 20HY vs 20NO but may still be considered as being reduced reaction genes. They are only significantly affected ($P_{adj} < 0.05$) in the 10HY vs 10NO comparison but not 20HY vs 20NO.

Correlating genes with metabolic performance

We used the method of Veilleux *et al.*, (2015) to investigate correlations of frontloaded genes with metabolism. Batch-corrected variance stabilised counts of frontloaded genes were correlated with MO_2. MO_2 was standardised as the residuals from a regression line between mass and MO_2 for the control group (10NO) (Fig. A5).

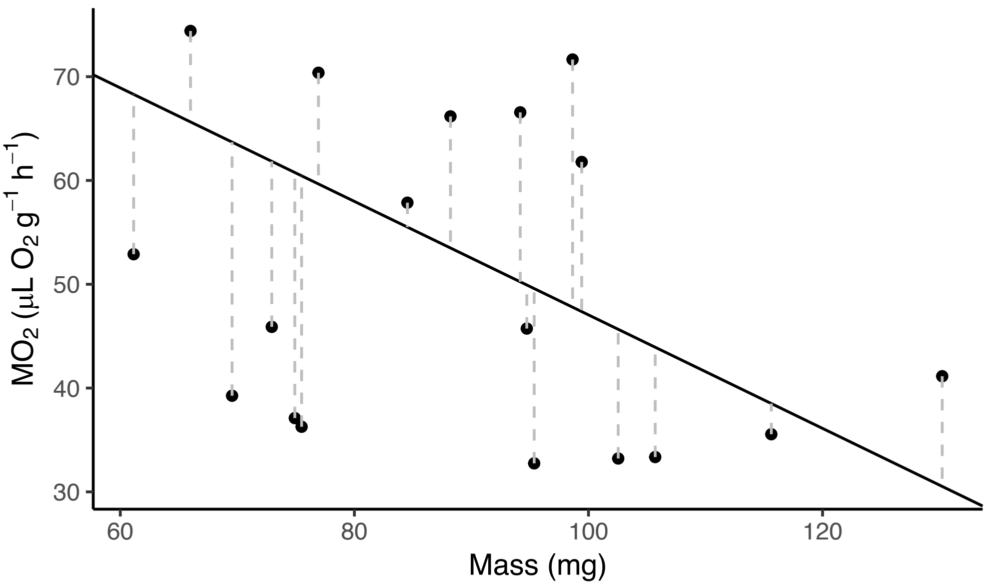

Fig. A5 Calculation of standardised MO_2. MO_2 was standardised as the residuals from the regression between mass and MO_2 for the cold acclimated normoxic control group, 10NO (MO_2 = -0.55 mass + 101.73, standardised MO_2 values in appendix C).

Supplementary Results

Transcriptome Assembly

The assembled transcriptome of *E. marinus* consisted of 383,395 transcripts assigned to 252,460 genes. N50 based upon the longest isoform per gene equalled 1114 bp. The E90N50 value equalled 2449 bp and the shape of the profile peaking at around ~90% suggests that sequencing was deep enough (Fig. A6). 79.55% of paired-end reads aligned concordantly against the transcriptome indicating a good

quality *de novo* assembly. BUSCO revealed 96.6% of arthropod orthologs were complete, 2% were fragmented and 1.4% missing (Fig. A6). Of the assembled transcripts, 114,497 and 66,953 were annotated against the Uniprot/Swissprot and PFAM databases respectively. 115,716 transcripts received gene ontology annotations (assembly and annotation statistics summarised in Appendix C).

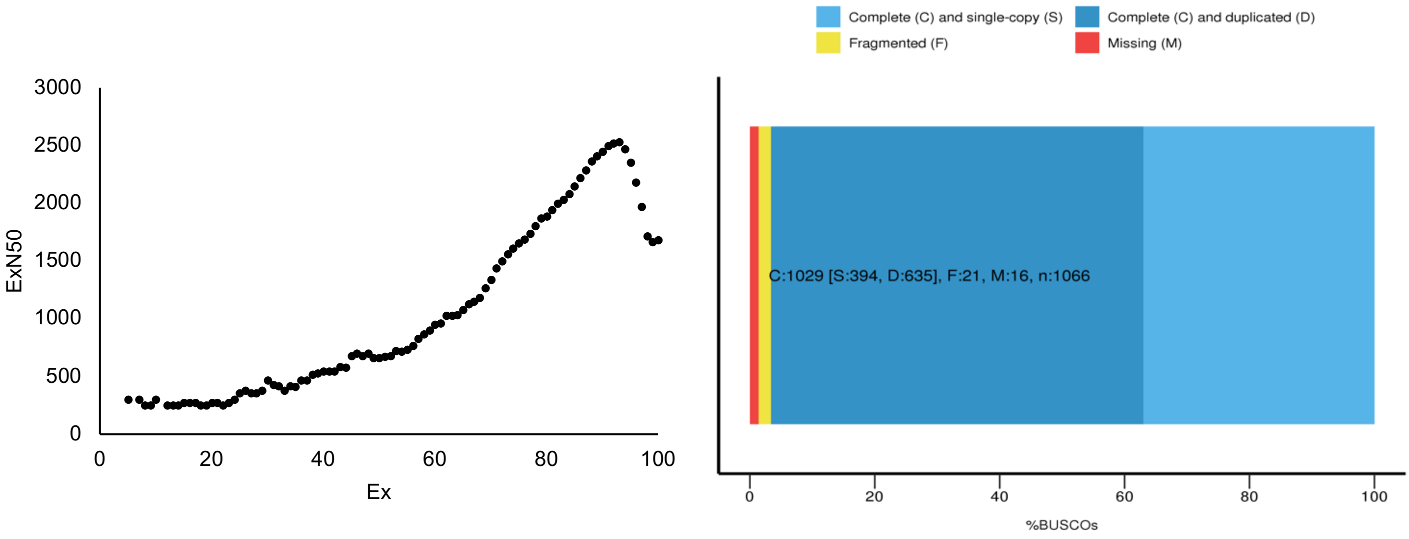

Fig. A6 Transcriptome assembly metrics. ExN50 and BUSCO assessment results plot (Waterhouse *et al.*, 2017) based upon all transcripts in the assembly.

Gene expression analyses

Following the differential expression analysis, biplots of principal components 1-5 were generated (Fig. A7). Only scores of PC1 and PC3 were significantly affected ($P < 0.05$) by treatment. To visualise the gene expression data, volcano plots of all genes were generated (Fig. A8). For the frontloaded genes, boxplots of genes belonging to the most significantly enriched GO terms are presented (Fig. A9 and Fig. A10).

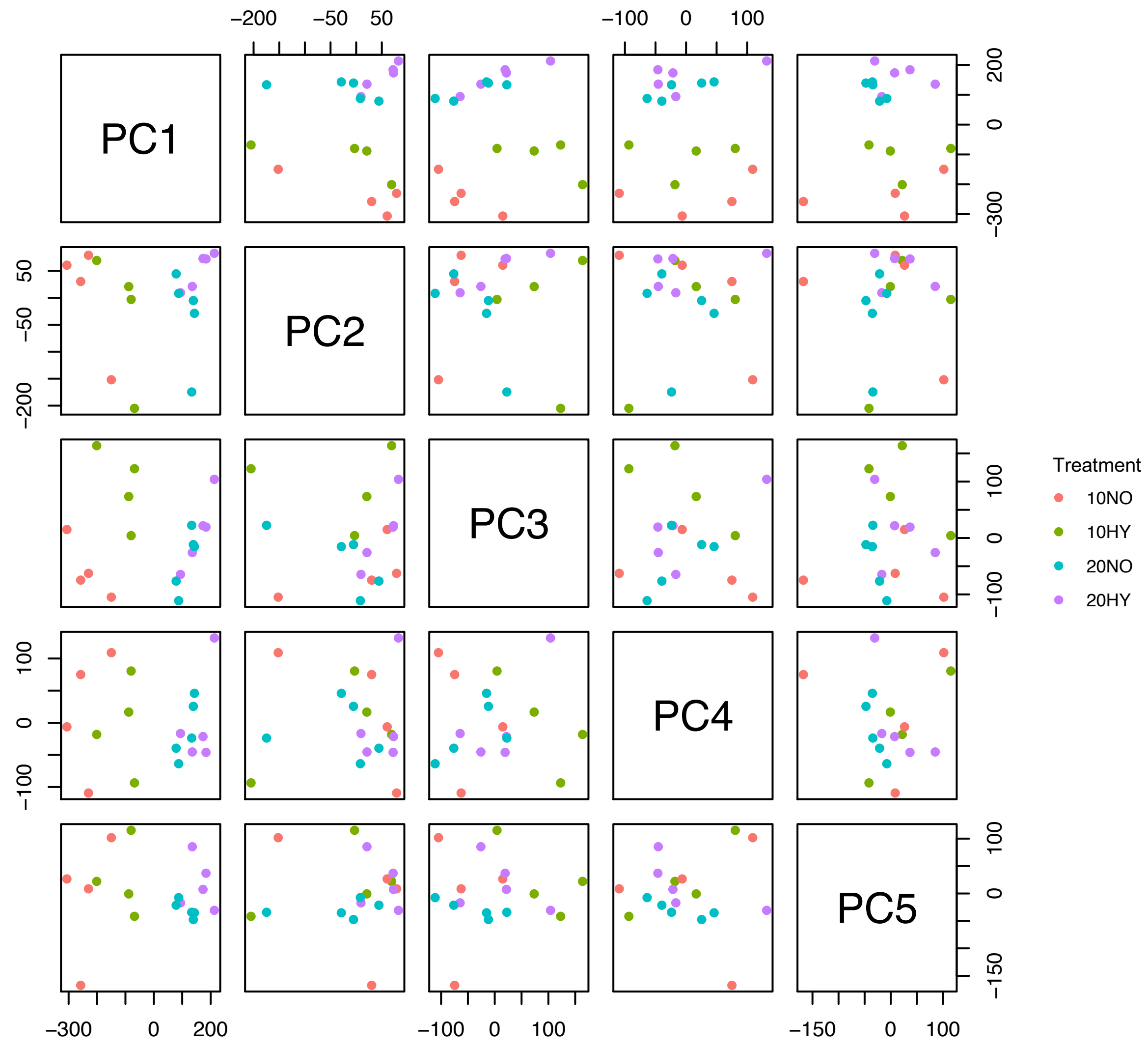

Fig. A7 PCA biplot of PC1-PC5. Based upon prcomp() of batch-corrected variance stabilised counts. Only PC1 and PC3 were significantly affected by treatment.

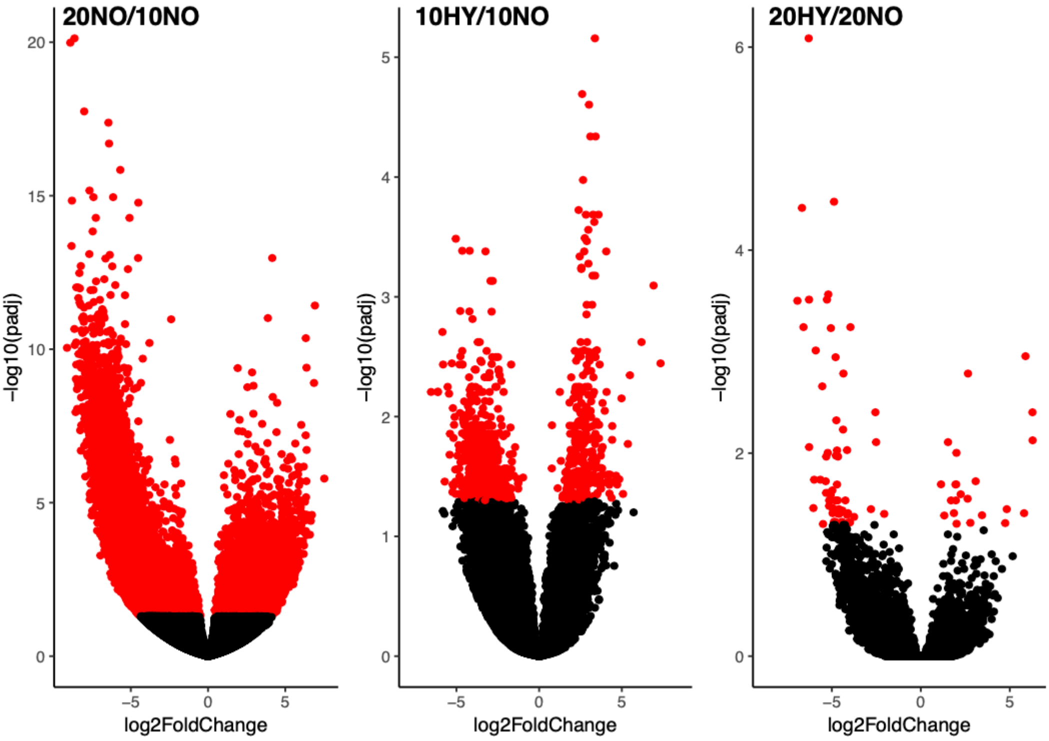

Fig. A8 Volcano plots of log2FC versus adjusted p-values for all genes. Red dots indicate significantly affected DEGs ($P_{adj} < 0.05$: 27,366 for 20NO vs 10NO, 1033 for 10HY vs 10NO, 88 for 20HY vs 20NO).

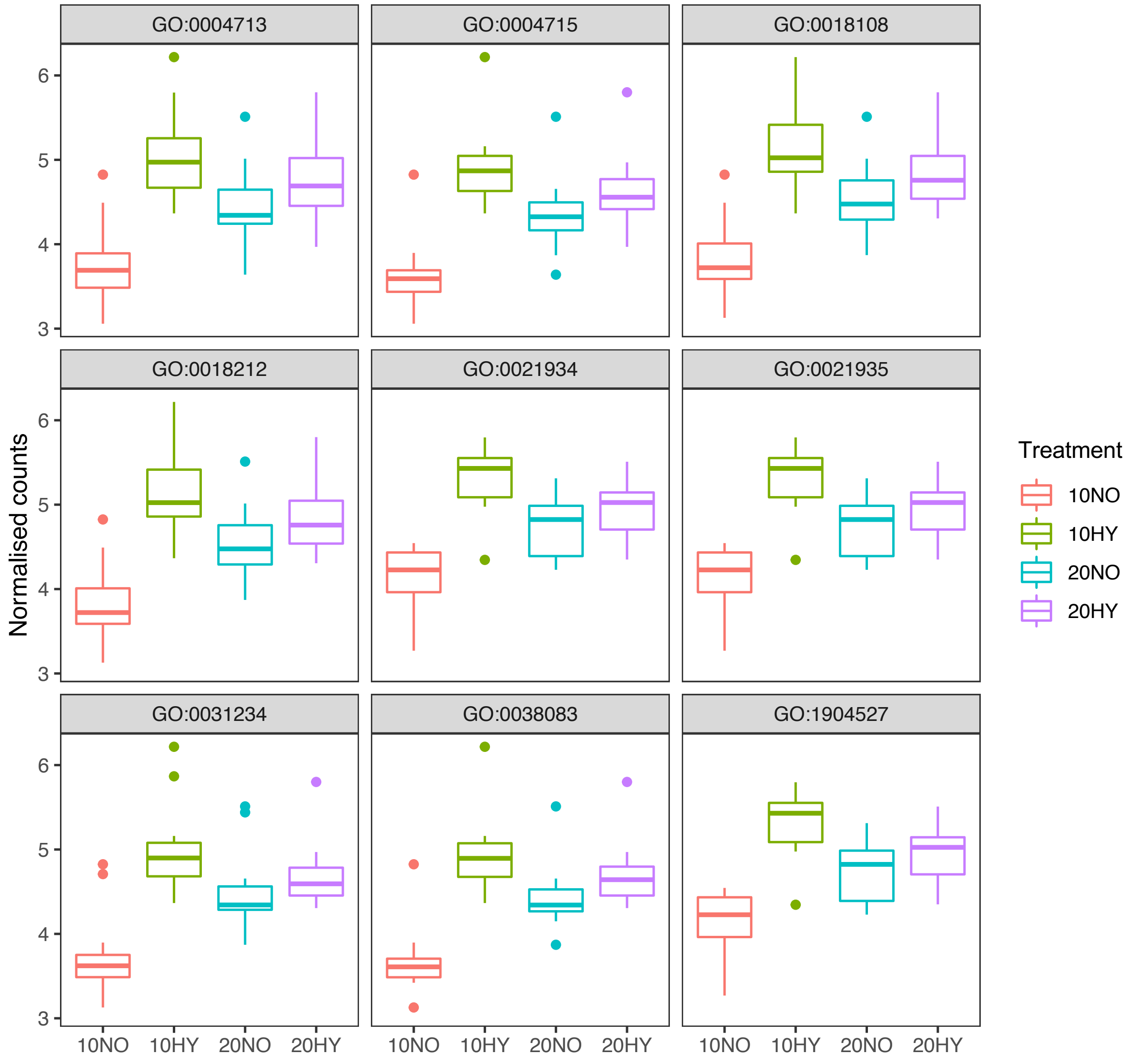

Fig. A9 Boxplot of variance stabilised counts for upregulated frontloaded genes belonging to the most significantly enriched GO terms. These genes are considered to be frontloaded as they show a reduced reaction to hypoxia in the warm acclimated compared to cold acclimated group (20HY vs 20NO fold upregulation < 10HY vs 10NO fold upregulation) which may be associated with higher constitutive expression resulting from thermal acclimation under normoxic conditions (20NO > 10NO). GO:0004713 protein tyrosine kinase activity, GO:0004715 non-membrane spanning protein tyrosine kinase activity, GO:0018108 peptidyl-tyrosine phosphorylation, GO:0018212 peptidyl-tyrosine modification, GO:0021934 hindbrain tangential cell migration, GO:0021935 cerebellar granule cell precursor tangential migration, GO:0031234 extrinsic component of cytoplasmic side of plasma membrane, GO:0038083 peptidyl-tyrosine autophosphorylation, GO:1904527 negative regulation of microtubule binding (full GO term results, Appendix D).

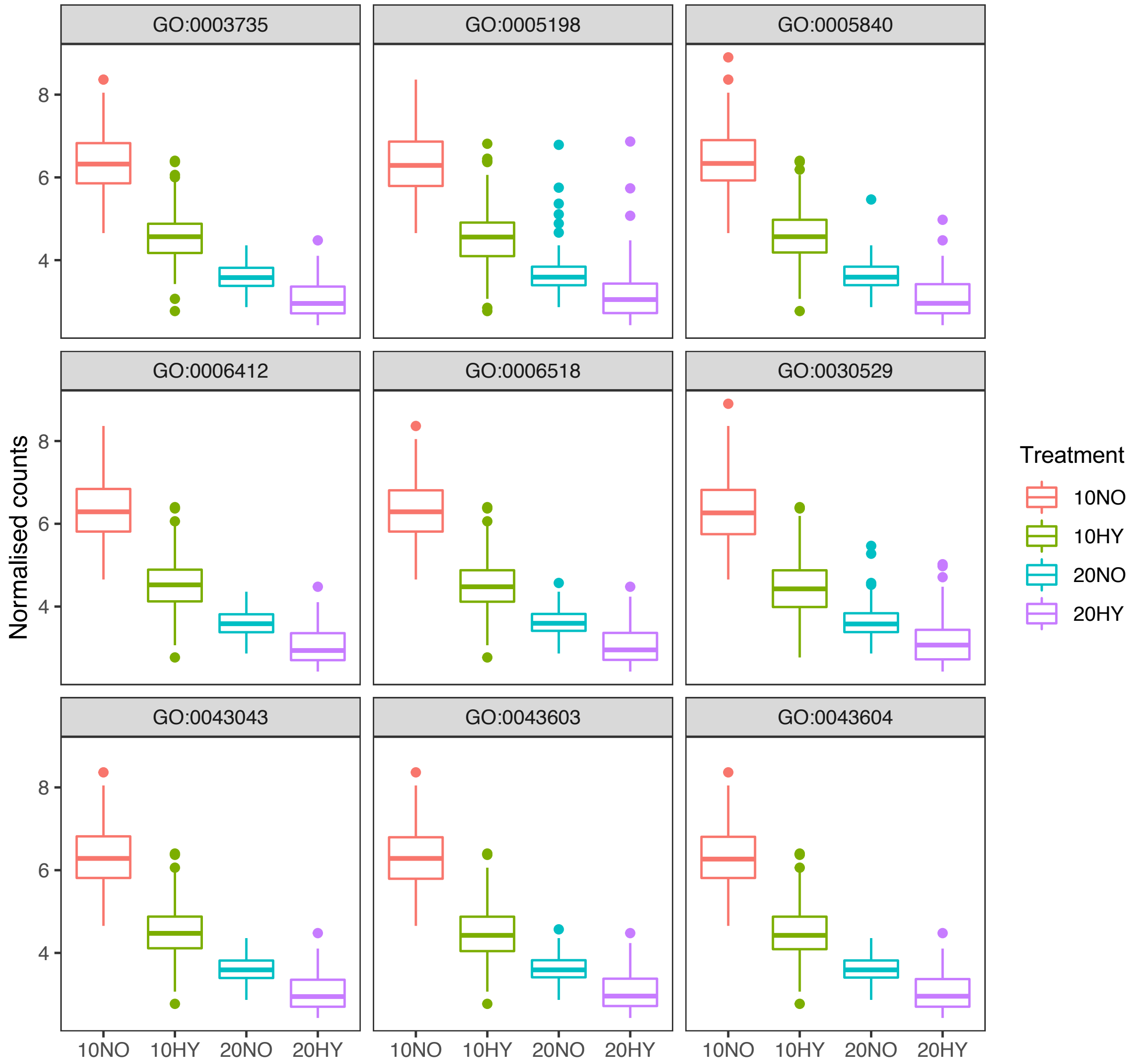

Fig. A10 Boxplot of variance stabilised counts for downregulated frontloaded genes belonging to the most significantly enriched GO terms. These genes are considered to be frontloaded as they show a reduced reaction to hypoxia in the warm acclimated compared to cold acclimated group (20HY vs 20NO fold downregulation < 10HY vs 10NO fold downregulation) which may be associated with lower constitutive expression resulting from thermal acclimation under normoxic conditions (20NO < 10NO). GO:0003735 structural constituent of ribosome, GO:0005198 structural molecule activity, GO:0005840 ribosome, GO:0006412 translation, GO:0006518 peptide metabolic process, GO:0030529 intracellular ribonucleoprotein complex, GO:0043043 peptide biosynthetic process, GO:0043603 cellular amide metabolic process, GO:0043604 amide biosynthetic process (for full GO term results, Appendix D).

References

Barshis, D. J., Ladner, J. T., Oliver, T. A., Seneca, F. O., Traylor-Knowles, N. and Palumbi, S. R. (2013) ‘Genomic basis for coral resilience to climate change’, *Proceedings of the National Academy of Sciences*, 110, pp. 1387–1392. doi: 10.1073/pnas.1210224110.

Bolger, A. M., Lohse, M. and Usadel, B. (2014) ‘Trimmomatic: a flexible trimmer for Illumina sequence data’, *Bioinformatics*, 30, pp. 2114–2120. doi: 10.1093/bioinformatics/btu170.

Haas, B. J., Papanicolaou, A., Yassour, M., Grabherr, M., Blood, P. ., Bowden, J., Couger, M. B., Eccles, D., Li, B., Lieber, M., Macmanes, M. D., Ott, M., Orvis, J., Pochet, N., Strozzi, F., Weeks, N., Westerman, R., William, T., Dewey, C. N., Henschel, R., LeDuc, R. D., Friedman, N. and Regev, A. (2013) ‘De novo transcript sequence reconstruction from RNA-Seq: reference generation and analysis with Trinity’, *Nature Protocols*, 8, pp. 1494–1512. doi: 10.1038/nprot.2013.084.

Huey, R. B. and Berrigan, D. (1996) ‘Testing evolutionary hypotheses of acclimation’, in Johnston, I.A. and Bennett, A. F. (ed.) *Animals and Temperature: Phenotypic and Evolutionary Adaptation*. Cambridge University Press, pp. 205–237.

Langmead, B. and Salzberg, S. (2012) ‘Fast gapped-read alignment with Bowtie 2’, *Nature methods*, 9, pp. 357–359. doi: 10.1038/nmeth.1923.

Li, H., Handsaker, B., Wysoker, A., Fennell, T., Ruan, J., Homer, N., Marth, G., Abecasis, G., Durbin, R. and Subgroup, 1000 Genome Project Data Processing (2009) ‘The Sequence Alignment/Map format and SAMtools’, *Bioinformatics*, 25, pp. 2078–2079. doi: 10.1093/bioinformatics/btp352.

Muggeo, V. M. R. (2008) ‘Segmented: an R package to fit regression models with broken-line relationships’, *R news*, 8, pp. 20–25.

Ritchie, M. E., Phipson, B., Wu, D., Hu, Y., Law, C. W., Shi, W. and Smyth, G. K. (2015) ‘Limma powers differential expression analyses for RNA-sequencing and microarray studies’, *Nucleic Acids Research*, 43, p. e47. doi: 10.1093/nar/gkv007.

Spicer, J. I. and El-Gamal, M. M. (1999) ‘Hypoxia accelerates the development of respiratory regulation in brine shrimp - but at a cost’, *Journal of Experimental Biology*, 202, pp. 3637–3646.

Todgham, A. E., Schulte, P. M. and Iwama, G. K. (2005) ‘Cross-tolerance in the tidepool sculpin: the role of heat shock proteins’, *Physiological and Biochemical Zoology*, 78, pp. 133–144. doi: 10.1086/425205.

Veilleux, H. D., Ryu, T., Donelson, J. M., Van Herwerden, L., Seridi, L., Ghosheh, Y., Berumen, M. L., Leggat, W., Ravasi, T. and Munday, P. L. (2015) ‘Molecular processes of transgenerational acclimation to a warming ocean’, *Nature Climate Change*, 5, pp. 1074–1078. doi: 10.1038/nclimate2724.

Verberk, W. C. E. P., Leuven, R. S. E. W., van der Velde, G. and Gabel, F. (2018) ‘Thermal limits in native and alien freshwater peracarid Crustacea: The role of habitat use and oxygen limitation’, *Functional Ecology*, 32, pp. 926–936. doi: 10.1111/1365-2435.13050.

Waterhouse, R. M., Seppey, M., Simao, F. A., Ioannidis, P., Klioutchnikov, G., Kriventseva, E. V. and Zbodnov, M. (2017) ‘BUSCO Applications from Quality Assessments to Gene Prediction and Phylogenomics’, *Mol Biol Evol*, 35, pp. 543–548. doi: 10.1093/molbev/msx319.
